# Near-complete genome sequencing of the feline coronavirus serotype I strain FIPV-Aqua from a cat with feline infectious peritonitis in Japan

**DOI:** 10.1128/mra.00357-25

**Published:** 2025-05-20

**Authors:** Yoshikazu Tanaka, Eri Tanabe, Takashi Sasaki

**Affiliations:** 1 Department of Veterinary Hygiene, Veterinary School, Nippon Veterinary and Life Science University 12989, Musashino, Tokyo, Japan; 2 Animal Research Center, Sapporo Medical University School of Medicine 92187, Sapporo, Japan; Queens College Department of Biology, Queens, New York, USA

**Keywords:** feline coronavirus type I, FIPV

## Abstract

Feline coronavirus causes the fatal disease feline infectious peritonitis (FIP), for which no effective vaccine is currently available. Here, we present the near-complete genome sequence of the Japanese strain FIPV-Aqua.

## ANNOUNCEMENT

Feline infectious peritonitis (FIP) is a fatal systemic granulomatous disease caused by feline coronavirus (FCoV), among the genus *Alphacoronavirus* in the family *Coronaviridae* (1). FCoVs are classified into serotypes I and II based on virus-neutralizing antibodies (2), with serotype I predominating in Japan (3). Understanding FCoV genetic diversity is key to vaccine developments for FIP; however, few studies have applied whole-genome sequencing to molecular epidemiology. We report the genome sequence of FCoV strain FIPV-Aqua from a cat with FIP in Japan.

The FCoV strain, FIPV-Aqua, was isolated from the pleural fluid of a 5-month-old male American shorthair cat. The patient presented with clinical signs suggestive of FIP. The FCoV gene was identified in the pleural effusion by reverse transcription quantitative PCR (4). Based on the observed symptoms and clinical biochemical findings, the cat was diagnosed with FIP. Samples were collected for clinical diagnosis of FIP, and institutional ethics approvals were not required for this work.

FCoV particles were isolated from fcwf-4 CU cells (5) infected with the pleural fluid of the cat. Total RNA was extracted from the cells using Isogen (Nippon Gene, Japan). The library was prepared using the Illumina Stranded mRNA Prep Ligation Kit (Illumina, USA). Sequencing was performed using a paired-end 2 × 151 bp cycle run on an Illumina NovaSeq 6000 system. We obtained a total of 162,760,371 reads; quality control and adapter trimming were performed by bcl-convert v3.9.3 (https://emea.support.illumina.com/sequencing/sequencing_software/bcl-convert.html). Using CLC Genomics Workbench v11 (Qiagen, Netherlands), after mapping to the genome sequence of *Felis catus* strain Fca126 (JAFEKA000000000.1) using 10% of trimmed reads, *de novo* assembly of the viral genome was performed using unmapped reads. To obtain the 5′ and 3′ terminal sequences of the viral genome, consensus sequences were generated by mapping analysis against the genome sequence of FCoV strain UU9 (FJ938062.1). Consequently, we generated a near-complete FCoV sequence without gaps. Gene annotation was performed based on the results of BLASTN analysis (https://blast.ncbi.nlm.nih.gov/Blast.cgi) against the reference FCoV serotype-I strains UU9 and C1Je (DQ848678) (6). Default parameters were used for all software unless otherwise specified.

**Fig 1:**
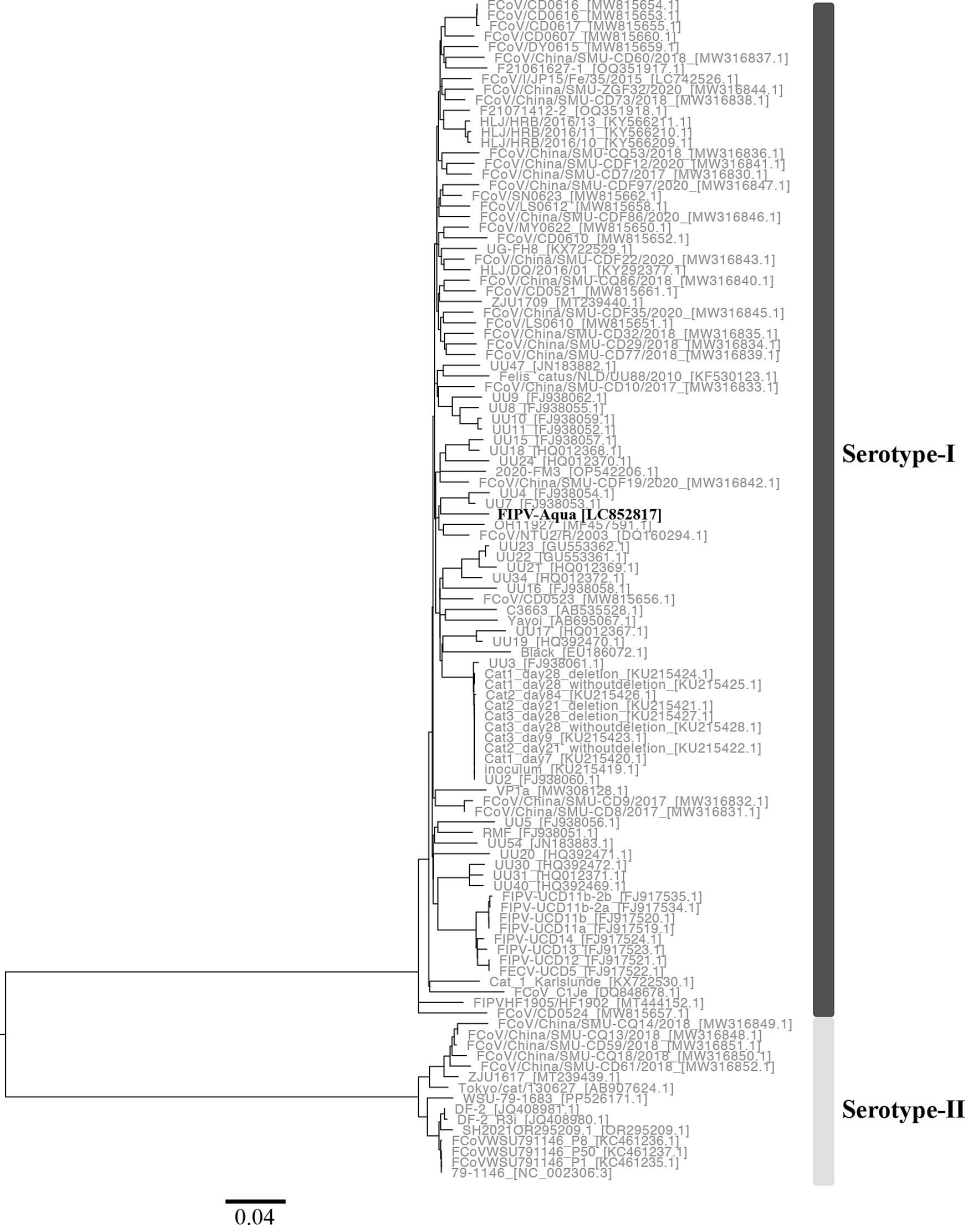
Phylogenetic tree based on 111 full-length amino acid sequences of FCoV spike protein was constructed by the neighbor-joining method. Strain FIPV-Aqua in the present study and those obtained from the NCBI database were indicated by black and grey letters, respectively. GenBank accession numbers were shown within square brackets. FIPV-Aqua was clustered into the clade containing serotype I strains, but was not closely related to any strains.

The FIPV-Aqua strain was phylogenetically identified as serotype I based on amino acid sequences of spike protein using MEGA v7 (Fig. 1) (7), with a genome size and G+C content of 28,522 bp and 38.39%, respectively. This genome contains 11 open reading frames encoding seven nonstructural proteins (polyprotein 1ab, polyprotein 1a, and nonstructural proteins 3a, 3b, 3c, 7a, and 7b) and four structural proteins (spike, envelope, membrane, and nucleocapsid). The polyprotein 1ab and 1a genes encode 16 nonstructural proteins (nsp1 to nsp16). Overlapping regions between these two genes, as well as a slip-site sequence (5′-UUUAAAC-3′) within them, were identified, consistent with typical coronavirus strains (6). The spike gene of this strain exhibited a deletion at the 5′ end, resembling those observed in FCoV strains such as UU16 (FJ938058.1), UU21 (HQ012369.1), Cat_1_Karlslunde (KX722530.1), and HF1902 (MT444152.1).

This entry of FCoV strain FIPV-Aqua genome into GenBank will contribute to various studies, including genomics, ecology, and epidemiology of FIP and FCoV.

## Data Availability

The raw WGS reads and the complete genome of the FCoV strain FIPV-Aqua obtained in this study have been deposited in DDBJ under the accession numbers DRR619600 and LC852817, respectively.
